# Supplementary material for: A microRNA negative feedback loop downregulates vesicle transport and inhibits fear memory
Source: eLife. 2016 Dec 21;5:e22467. doi: 10.7554/eLife.22467 (PMC5293492; doi:10.7554/eLife.22467)
Supplement: Supplementary file 1. — (A) List of 353 targets with predicted binding sites for 3 or more of the fear-induced miRNAs. (B) List of targets from the vesicle exocytosis pathway with predicted binding sites for 3 or more fear-induced miRNAs. (C) Predicted Targets of miR-153 from top five Metacore networks. DOI: http://dx.doi.org/10.7554/eLife.22467.024 [file elife-22467-supp1.pdf]

Supplementary file 1A. List of 353 targets with predicted binding sites for 3 or more of the fear-induced miRNAs.

<u>Gene symbols</u>

0610007L01Rik	Bcl2l11	E2f3	Hipk3	Mllt3	Rbm47	Socs7	Ypel2
1110067D22Rik	Bcl7a	Ebf3	Hlf	Msi2	Rc3h1	Sos1	Ythdf2
1200014J11Rik	Bend3	Efnb2	Hmga2	Mtmr12	Ret	Sp1	Yy1
1300001I01Rik	Birc6	Eif2c3	Hnrnpa2b1	Naa15	Rfx3	Specc1l	Zbtb34
4933433P14Rik	Bmi1	Eif4e3	Hs3st3b1	Nbea	Rfx7	Spire1	Zbtb39
A930001N09Rik	Braf	Elavl4	Igf2bp1	Nedd4l	Rgs7bp	Spty2d1	Zbtb41
Aak1	Brpf3	Ell2	Igf2bp3	Nfat5	Rlim	St8sia4	Zcchc14
Abl2	Bsn	Eml5	Ikzf4	Nfia	Rmnd5a	Strbp	Zdhhc20
Accn2	C130039O16Rik	En2	Inhbb	Nfib	Rnf150	Stxbp5	Zdhhc21
Acsl4	C77370	Epc2	Ino80	Nhlh2	Rnf165	Synj1	Zdhhc5
Adam23	Camk4	Epha7	Ipo8	Nlk	Rnf38	Sypl	Zeb2
Adamts3	Camta1	Esrrg	Kcnh1	Nmnat2	Robo2	Tada2b	Zfhx4
Adipor2	Capza1	Ets1	Kcnmb2	Nova1	Rorb	Tanc2	Zfp236
Afap1	Cask	Faf2	Kcnq5	Nufip2	Rtf1	Taok1	Zfp238
Agap1	Cbfa2t3	Fam126b	Kif1b	Onecut2	Runx1	Tbc1d8	Zfp36l1
Agps	Cbx5	Fam178a	Klf12	Osbpl3	Runx1t1	Tcf4	Zfp385c
Ak4	Ccdc6	Fam196a	Klhl29	Osbpl6	Runx2	Tet3	Zfp462
Amot	Ccdc85a	Fam196b	Lats1	Palm2	Sall4	Tgfbr2	Zfp609
Ank3	Ccdc88a	Fat3	Lhfp	Pbrm1	Samd8	Thoc2	Zfp697
Ankrd13b	Cdk8	Fbln5	Lin28b	Pclo	Scai	Thsd7a	Zfp704
Ankrd52	Cecr2	Fbxl17	Lin7a	Pdcd6ip	Scn3a	Timp2	Zfyve20
Ap4e1	Chic1	Fbxo28	Lin7c	Pde7a	Scn8a	Tmem33	Zmynd11
Apba1	Clock	Fbxo33	Lmx1a	Pdgfra	Sdc2	Tmtc2	Znrf3
Api5	Cnot2	Fgf9	Lphn1	Phactr2	Sema6a	Tnrc6b	Zzz3
Appl1	Cnr1	Flrt3	Lpp	Phf13	Sept11	Tox3	
Arcn1	Cntn4	Fmr1	Lrba	Phf15	Sept8	Tpm3	
Arf6	Cpeb2	Foxp2	Maf	Phf20l1	Serinc5	Traf3	
Arhgef12	Ctdspl	Frmd6	Magi2	Pi4k2a	Setbp1	Trim33	
Arid1b	D3Bwg0562e	Fsd1l	Man2a1	Pip4k2b	Setd7	Trim71	
Arid4b	D430041D05Rik	Fyco1	Map3k2	Plcl2	Sfmbt1	Trip12	
Arx	D630045J12Rik	Gab2	Mapk10	Ppargc1a	Sgms2	Trps1	
Asph	Dcbld2	Gabrb2	Mapre1	Ppfia2	Sh3pxd2a	Tsc22d2	
Asxl3	Dcc	Gdnf	Mar4	Prickle2	Shoc2	Ttbk2	
Atf2	Dclk1	Glcci1	Marcks	Prrx1	Sik1	Ttc14	
Atg14	Dcp1a	Glce	Mast4	Psd2	Sirt1	Ube2h	
Atp2b1	Dcp2	Gli3	Mbnl1	Pten	Slc12a2	Ube2i	
Atp8b2	Dcun1d3	Gm2058	Mcc	Ptp4a1	Slc16a10	Ubfd1	
Atrn	Dcx	Gnaq	Mdga2	Ptprg	Slc22a23	Ubn2	
Atxn1	Ddx3x	Gng12	Mecp2	Qk	Slc24a2	Ubxn7	
B230219D22Rik	Derl2	Gpr153	Med12l	Qser1	Slc30a7	Unc13a	
B630005N14Rik	Dlg2	Grik2	Mef2c	Rab15	Slc38a4	Unkl	
Bach2	Dnajc3	Grik3	Mfap3l	Rab40b	Slc39a1	Usp31	
Bahd1	Dnalc1	Grm1	Mga	Rap1b	Slc39a9	Usp42	
Baz2a	Dnmt3a	Gse1	Mier3	Rapgef2	Slc7a11	Vat1	
Bcl11a	Dnmt3b	Hapln1	Mink1	Rasgrf2	Slc8a1	Vav3	
Bcl11b	Dolpp1	Has3	Mll1	Rassf10	Smoc2	Wipf1	
Bcl2	Dpysl2	Hic2	Mll2	Rbfox2	Socs6	Wipi2	

Supplementary file 1B. List of targets from the vesicle exocytosis pathway with predicted binding sites for 3 or more fear-induced miRNAs. The miRNAs that co-regulate each gene are listed underneath the gene name.

**Apba1**	**Bsn**	**Erc1**	**Pclo**	**Ppfia2**
let-7f	let-7f	miR-29b	miR-27b	miR-15a
miR-9	miR-30e	miR-29c	miR-153	miR-27b
miR-30e	miR-153	miR-130a	miR-203	miR-30e
	miR-218		miR-218	miR-130a

**Rph3al**	**Slc1a2**	**Snap25**	**Stx1a**	**Stxbp5**
miR-15a	miR-30e	miR-27b	miR-9	let-7f
miR-130a	miR-153	miR-130a	miR-27b	miR-30e
miR-153	miR-203	miR-153	miR-29b	miR-218
miR-218			miR-29c	

**Syt1**	**Syt4**	**Trak2**	**Vamp1**	**Vamp3**
let-7f	miR-9	miR-29	miR-9	miR-9
miR-153	miR-15	miR-153	miR-15a	miR-30e
miR-218	miR-153	miR-181a	miR-181a	miR-181a
		miR-181b	miR-181b	miR-181b
				miR-338-3p

Supplementary file 1C. List of targets from each of the top five networks (axon guidance, long-term potentiation, synaptic contact, synaptogenesis, and vesicle exocytosis pathways) with predicted binding sites for miR-153. Genes from the vesicle exocytosis pathway that are co-regulated by miR-153 together with other fear-induced miRNAs are presented in Figure 1E.

**Axon Guidance**	**LTP**	**Synaptic Contact**	**Synaptogenesis**	**Vesicle Exocytosis**
Adam23	Src	Cask	Bsn	Bsn
Calm1	Calm1	Dlg2	Cask	Cltc
Cacna1c	Grb2	Efnb2	Dlg2	Itsn2
Dcc	Pik3r1	Fyn	Efnb2	Pclo
Efnb2	Prkar2b	Grik2	Grik2	Rph3al
Fyn	Plcb1	Grip2	Grik3	Slc1a2
Prkar2b	Ryr2	Nfasc	Scn8a	Snap25
Robo2	Ryr3	Snap25	Pclo	Snca
Ryr2		Wipf1		Syt1
				Syt4
				Trak2
				Vamp2
